# Role of Phosphorylation in the Control of Clathrin-Mediated Internalization of GPCR

**DOI:** 10.1155/2011/246954

**Published:** 2011-06-07

**Authors:** Frederic Delom, Delphine Fessart

**Affiliations:** ^1^Bordeaux Cardiothoracic Research Center, Bordeaux University, 146, Léo-Saignat, 33076 Bordeaux, France; ^2^Inserm U1045, 146, Léo-Saignat, 33076 Bordeaux, France

## Abstract

The process by which G protein-coupled receptors (GPCRs) are internalized through the clathrin-coated vesicles involves interactions of multifunctional adaptor proteins. These interactions are tightly controlled by phosphorylation and dephosphorylation mechanisms resulting in the regulation of receptor endocytosis. However, the identities of the kinases involved in this process remained largely unknown until recently. This paper discusses advances in our knowledge of the important role played by protein phosphorylation in the regulation of the endocytic machinery and how phosphorylation controls the coated vesicle cycle.

## 1. An Overview on G Protein-Coupled Receptors (GPCRs) Internalization

Reversible protein phosphorylation by the interplay between kinases and phosphatases is a major regulatory mechanism for G protein-coupled receptors (GPCRs) internalization [1–3]. Therefore, the aim of this paper is to synthesise our understanding of the phosphorylation mechanisms regulating GPCRs internalization. Growing interest in this field is due to the involvement of GPCRs in the regulation of a number of functions. Following their activation, these receptors enter inside the cells, a process named receptor internalization. This process requires receptor desensitization which is achieved by receptor phosphorylation, sequestration and internalization. In this manner, receptors are removed from the surface and transferred into cells. Inside the cells, receptors are embedded into small membrane vesicles (endosomes) which may be recycled back to the plasma membrane in order to renew their fully functional coupling with proteins and effectors (i.e., resensitization). GPCRs expression can also be regulated by a process of downregulation which is a terminal stage of receptor life where receptors are degraded in lysosomes. However, it is now becoming apparent that GPCRs can also be regulated independently of their phosphorylation state (reviewed in [4]). Before starting the role played by phosphorylation during receptor internalization, a brief review of the different steps controlling receptor internalization will be given.

### 1.1. Steps Conducting to Receptor Internalization

**Figure 1:**
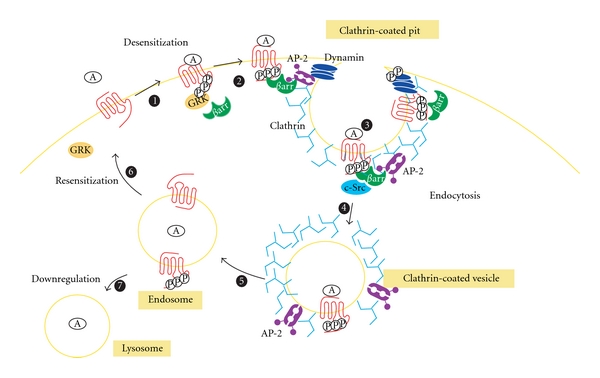
Steps conducting to G protein coupled receptors internalization. Internalization and recycling of GPCRs. Upon agonist binding, receptors are phosphorylated (P) by GRKs leading to the recruitment of *β*-arrestins (step 1). Beta-arrestins, through their interaction with clathrin and AP-2, target the receptor/arrestin complexes to clathrin coated pits (step 2). Beta-arrestins also bind c-Src (steps 2 and 3). Dynamin (step 3), a GTPase, regulates the pinching off from the cell surface of clathrin-coated pits (steps 3 and 4). Once clathrin-coated vesicles are formed (step 4), the receptor is then internalized into endosomes (step 5), dephosphorylated before returning to the cell surface (step 6), or the receptor is degradated to lysosomes (step 7).

The current proposed model for GPCRs internalization is essentially based on *β*2-adrenergic receptor (*β*2-AR) studies. Briefly, following agonist stimulation, GPCRs undergo conformational change that allow binding of G proteins, leading to the activation of different effectors and signaling pathways [5]. The desensitization process is then activated (Figure 1). One of the first steps involves the functional “uncoupling” of the G proteins from the receptors. The receptor is subsequently phosphorylated by G protein-coupled receptor kinases (GRKs) to enhance the binding of *β*-arrestins (step 1, Figure 1).*β*-arrestins act as scaffolding intermediates with components of the clathrin-coated-pit machinery, thus stabilizing association with clathrin-coated pits (CCPs) (steps 2 and 3, Figure 1). The formed pits progressively invaginate and are finally released into the cytosol as a free clathrin-coated vesicle (CCV) (step 4, Figure 1), a step which requires the GTPase activity of dynamin to pinch off clathrin-coated vesicles from the plasma membrane [6]. After coat assembly and following vesicle scission, all the components must be disassembled so that the CCV can fuse with an early endosome (step 5, Figure 1). The early endosome controls the activity and the destination of proteins in the compartment. Therefore, endosomes are a key control point for sorting receptors, which can be directed to recycling endosomes and back to the cell surface (step 6, Figure 1), or directed to late endosomes or lysosomes for degradation (step 7, Figure 1). Even though several groups have studied GPCRs internalization via the clathrin-coated vesicles, a clear picture of all the phosphorylated proteins involved in this mechanism that regulates GPCRs internalization still remains elusive.

## 2. Regulation of GPCRs Internalization by Receptor Phosphorylation

Following receptor activation by an agonist, the receptor undertakes the process of desensitization, defined as “off switch” of all receptor functions triggered by the agonist at the plasma membrane. Therefore, GPCRs control their own responsiveness to dampen the physiological response to the continued presence of the stimulus [7]. This process is the consequence of a combination of different mechanisms. The cloning of the *β*2-AR in 1986 and its shared homology with the rhodopsin receptor [8] allowed the discovery that both receptors become phosphorylated in a stimulus-dependent way and that this phosphorylation seemed to be related to the process of receptor desensitization. Desensitization is thought to be mediated through phosphorylation of serine and threonine residues by G protein-coupled receptor kinases (GRKs) [9], second messenger-dependent protein kinases such as cAMP-dependent kinase (PKA), and protein kinase C (PKC) [10–14].

### 2.1. Receptor Phosphorylation by Second Messengers: Heterologous Desensitization

PKA and PKC are phosphotransferases that catalyze the transfer of the *γ*-phosphate group of ATP to serine and threonine residues contained within specific amino acid consensus sequences of proteins. They can phosphorylate a receptor even in the absence of its agonist, resulting in “heterologous” desensitization (reviewed in [15]). These kinases are activated in response to GPCRs-stimulated increases in intracellular second messengers, such as cAMP, Ca^2+^ and diacylglycerol (DAG), and participate in GPCRs signaling by mediating the phosphorylation of the receptor itself or other downstream target proteins. In the latter case, receptors that have not been activated by a ligand may become desensitized by activation of these kinases. PKA-mediated receptor phosphorylation has also been shown, in the case of the *β*2-AR, to switch coupling of the receptor away from G_s_ in favour of enhanced coupling to G_i_ [16]. Several other receptors, including the prostacyclin receptor, also seem to undergo such PKA-mediated switching in their G protein-coupling specificity [17]. The phosphorylation of GPCRs by PKA and PKC has also been shown for other receptors such as gamma-aminobutyric acid receptor type B (GABA_B_) [18, 19]. In addition to these kinases, the 5′AMP-dependent kinase (AMPK), a serine/threonine protein kinase, has been shown to phosphorylate GABA_B_ receptor on Ser-783 to further decrease receptor desensitisation [20].

### 2.2. Receptor Phosphorylation by G Protein-Coupled Receptor Kinases (GRKs): Homologous Desensitization

By the mid-1980s, it had become clear that both rhodopsin [21] and the *β*2-AR [22] were phosphorylated in a stimulus-dependent way, and that this phosphorylation seemed to be related to receptor inactivation or desensitization. In the case of rhodopsin, the enzyme responsible for the phosphorylation was referred to as rhodopsin kinase [23]. In the case of the *β*2-AR, a novel cAMP-independent kinase [called the *β*-adrenergic receptor kinase (*β*ARK)] appeared to contribute to agonist-dependent phosphorylation of the receptor [24]. Now, 7TM receptors (GPCRs) are traditionally thought to signal by means of activation of heterotrimeric G proteins and then to be desensitized by GRKs which are recruited to and specifically phosphorylate only agonist-occupied receptors leading to “homologous desensitization” [24, 25]. 

#### 2.2.1. GRK Family

GRKs phosphorylate GPCRs at both serine and threonine residues localized within the third intracellular loop (i3) or C-terminal tail domain [26]. Although no putative GRK phosphorylation consensus motifs have been identified, localization of amino acidic residues flanking repeated serines/threonines to the site of phosphorylation seems to favour GRK-2-mediated phosphorylation [27]. So far, only GRKs selectively phosphorylate agonist-activated receptors, whereas second messenger-dependent kinases can phosphorylate receptors in the presence or absence of an agonist. Seven mammalian GRK genes have been identified, some of which undergo alternative splicing to generate different isoforms [26, 28]. The GRKs consist of three distinct domains: the kinase domain, the N-terminal RGS (regulator of G protein signaling) domain, and the C-terminal domain which contributes to the plasma membrane targeting of the kinase. The function of RGS proteins is to inhibit the activity of the G*α*-subunit by acting as GTPase-activating proteins (GAPs) [29]. The specificity of GRKs *in vitro* has not yet been defined, but tissue distribution, as well as levels of expression, probably contributes to their specificity *in vivo* [30–32]. However, studies show that different GRKs induce distinct signaling upon Angiotensin II type 1 receptor (AT1R) or V2 vasopressin receptor (V2R) activation, and suggest that GRK-2 and -3 will antagonize the effects of GRK-5 and -6 [33, 34]. Several factors control the activity of the kinases towards the receptors. Principally, the activated conformations of the receptors themselves activate the enzymes [31].

#### 2.2.2. GRK Phosphorylation to Regulate its Own Activity

A regulatory complexity is added by the fact that GRK activity can be influenced by phosphorylation. Mitogen-activated protein kinase (MAPK) decreases its efficacy towards GPCRs substrates [35], whereas both PKA and PKC can phosphorylate and activate GRK-2 by promoting its G*βγ*-mediated membrane association [36, 37] and potentiating its activity [36, 38]. In contrast, GRK-5 activity can be influenced by phosphorylation of PKC kinase to reduce its activity [39]. Moreover, c-Src, via its recruitment to the agonist-dependent binding of *β*-arrestin to GPCRs, phosphorylates GRK-2 on tyrosine residues and targets GRK-2 for degradation [40, 41]. 

#### 2.2.3. GRK Phosphorylation of Receptors for *β*-Arrestin Recruitment

GRK-phosphorylation of receptors is not sufficient for desensitization, but rather serves to create high affinity sites to promote the recruitment of the cytoplasmic accessory proteins, arrestins, and target the receptors for internalization via clathrin-coated pits. The arrestin protein was first identified by Wilden et al. in 1986 as, “a 48K protein”, bound to the phosphorylated rhodopsin, thereby interfering with its coupling to transducin [42]. The protein was later renamed arrestin [43]. Therefore, it was postulated that the GRK-mediated phosphorylation of clusters of serine and threonine residues in the C-terminal tails of some receptors may regulate the stability of receptor/arrestin complexes [44]. For most receptors, the determining factor for *β*-arrestin interaction is the phosphorylation status of the activated GPCRs. Thus, impairment of receptor phosphorylation by mutagenesis of key serine/threonine residues generally leads to diminished *β*-arrestin binding after stimulation of the M2 muscarinic cholinergic receptor, rhodopsin, the AT1R, or the V2R [45–48]. The arrestins recruitment in turn induces desensitization by preventing further coupling to G proteins [49, 50]. However, there are some exceptions and widespread variations against a general model of receptor phosphorylation in the regulation of internalization. For example, the findings that mutants of the parathyroid hormone receptor underwent endocytosis in the absence of detectable agonist-induced phosphorylation [51]. In addition, Richardson et al., [52] have shown that substance P receptor lacking the C-terminal domain remains competent to desensitize and internalize. These and similar data obtained with a truncated opioid receptor [53] or with the leukotriene B4 receptor 1 (BLT1) [54] do not support a general role for receptor phosphorylation in the control of GPCRs internalization. Overall, these findings led Kim et al. to suggest that the role of receptor phosphorylation allow the access of arrestin to its receptor binding domain than to create an arrestin-binding site [55].

### 2.3. Receptor Phosphorylation by Casein Kinase II (CK2)

Casein kinase II has been reported to phosphorylate the M3–muscarinic receptor in cerebella granule neurons and to affect coupling to the Jun-kinase pathway [56]. It was also suggested that 3 putative CK2 phosphorylation sites on the carboxyl tail of the TSH-releasing hormone receptor (TRHR) were important for its internalization [57]. In addition to TRHR receptor, the Leukotriene B4 receptor (G*α*_o_-coupled) contains also a putative CK2 site, which, when mutated, reduced GRK-6-mediated desensitization [58]. Rebholz et al. have suggested that GPCRs regulation by CK2 is exerted through its ability to enable faster endocytosis of G_s_-coupled receptors [59]. However, it remains unclear if this type of phosphorylation is common to others GPCRs family members.

## 3. Regulation of GPCRs Internalization by Arrestin Phosphorylation

Despite numerous studies suggesting an essential role of arrestins in GPCRs regulation, the detailed mechanism underlying the function of arrestins and receptor-mediated modulation of arrestin activity is unclear. *β*-Arrestin are component of a multiprotein complex that assembles on the receptor after ligand stimulation. Acting as a protein scaffold, it recruits Src kinase and also binds to clathrin, and adaptor-protein 2 (AP-2), which serves to target the activated GPCRs to clathrin-coated pits for internalization. *β*-Arrestin also induce rapid homologous desensitization of the receptor by binding to the GPCRs and sterically inhibiting further G-protein coupling. However, several reports on arrestin phosphorylation provide some insight into potential regulation of arrestin function. 

### 3.1. Arrestin Phosphorylation by Extracellular Signal-Regulated Kinases 1 and 2 (ERK1/2)

Studies on mammalian *β*-arrestin 1 demonstrate that *β*-arrestin 1 was constitutively phosphorylated at Ser-412 by extracellular signal-regulated kinases 1 and 2 and became dephosphorylated following *β*_2_AR stimulation [60, 61]. *β*-arrestin 1 phosphorylation was proposed to regulate the interaction of arrestin with clathrin and Src, since a S412D mutant mimicking phosphorylated *β*-arrestin 1 had reduced binding to these proteins [60, 62]. Thus, agonist treatment of GPCRs, which induces dephosphorylation of *β*-arrestin 1 at Ser-412, as well as transfection of the S412A *β*-arrestin 1 mutant, which mimics dephosphorylated *β*-arrestin 1 and enhances clathrin and Src binding to *β*-arrestin 1, resulting in enhanced GPCRs internalization. Notably, *β*-arrestin 2 shares ~80% amino acid identity with *β*-arrestin 1 but does not contain the corresponding serine residue. Thus, *β*-arrestin 2 might be phosphorylated at other sites or perhaps be regulated by different mechanisms. 

### 3.2. Arrestin Phosphorylation by GRK

Recently, *β*-arrestin 1 has also been shown to be phosphorylated at Ser-412 by GRK-5 [63]. This phosphorylation, in turn, prevents the activation of c-Src and, therefore, blocks ERK signalling.

### 3.3. Arrestin Phosphorylation by CK2

Kim et al. have reported that *β*-arrestin 2 is constitutively phosphorylated by casein kinase II at Thr-383 and suggested that *β*-arrestin 2 phosphorylation may regulate its interaction with novel binding partners unidentified [64]. Threonine 383 is the primary phosphorylation site, and serine 361 represents a secondary site [64, 65]. Dephosphorylation of *β*-arrestins at the plasma membrane seems to be necessary for engaging endocytic partners.

### 3.4. Arrestin Phosphorylation by Calcium/Calmodulin-Dependent Kinase II (CaMKII)

In *Drosophila,* two visual system arrestins have been identified: Arr1 and Arr2. Both Arr1 and Arr2 are phosphorylated in a light-dependent manner, and it has been established that Arr2 is phosphorylated by a Ca^2+^/calmodulin-dependent protein kinase (CaMKII) [66]. Although it has been known for quite some time that Arr2 is phosphorylated in a light-dependent manner, it is unclear just what role this phosphorylation serves. Studies with *Drosophila* visual arrestin-2 demonstrate that it undergoes light-dependent phosphorylation by calcium/calmodulin-dependent kinase II and that phosphorylation is necessary for dissociation of arrestin from rhodopsin [67, 68]. It has been proposed that the calcium- and light-dependent phosphorylation of Arr2 acts as the signal to bind and inactivate metarhodopsin, and therefore, Arr2 phosphorylation serves to modulate the inactivation of the signaling cascade [66, 69–71]. However, recent evidence suggest that the phosphorylation of Arr2 is also necessary for its release from membranes once rhodopsin has been photoconverted back to its inactive form [68].

### 3.5. Arrestin Phosphorylation by Src

Src kinases phosphorylates tyrosine residues on the clathrin coated pit structural proteins including clathrin itself and dynamin but also *β*-arrestin. There are four tyrosines present in the N-terminall part of *β*-arrestin-1 that are not conserved in *β*-arrestin-2 [72]. One of these residues is Tyr-54 which is phosphorylated by Src, and a single point mutation Y54F is sufficient to prevent its phosphorylation. The constitutive substitution of *β*-arrestin-1 tyrosine 54 by phenylalanine increases the *β*-arrestin-1 interaction with the *μ*-subunit of the AP-2 and enhances *β*-arrestin-1-mediated *β*2-AR internalization.

Furthemore, *β*-arrestin has been shown recently to mediate phosphorylation events by activating 38 protein kinases [73]. However, it remains unclear if this effect is correlated with the *β*-arrestin-phosphorylation status.

## 4. Regulation of GPCRs Internalization by Clathrin Phosphorylation

Clathrin is also subject to phosphorylation both *in vitro* and *in vivo* and are thought to serve as regulatory subunits [74, 75]. The very limited data on this suggest that the phosphorylation of clathrin could be inhibitory for coat assembly [76, 77]. 

### 4.1. Clathrin Phosphorylation by c-Src

Tyrosine phosphorylation of clathrin was shown only for the epidermal growth factor receptor. Tyrosine phosphorylation of the clathrin heavy chain by Src-family kinases enhances the clathrin recruitment to the plasma membrane. It was shown that ligand binding to the epidermal growth factor receptor leads, via activation of protein kinase activity, to clathrin phosphorylation [78].

### 4.2. Clathrin Phosphorylation by CK2

Casein kinase 2 plays an essential role in endocytosis, the inhibition of CK2 leads to a significant decrease in transferrin uptake [79]. CK2 is highly enriched in CCV preparations and phosphorylates the clathrin light chain *in vitro* [75]. As mentioned above, the clathrin light chain is also found to be phosphorylated *in vivo*, but the functional role of this phosphorylation event in endocytosis is not entirely clear.

## 5. Regulation of GPCRs Internalization by Adaptor Proteins (AP)

Clathrin-mediated endocytosis is the well-described mechanism for the entry of molecules into cells. This pathway is characterized by the recruitment of soluble clathrin from the cytoplasm to the plasma membrane. Clathrin-binding adaptors, such as the adaptor-protein 2 (AP-2), are the key components of this pathway. They bind directly to clathrin [80], as well as *β*-arrestin and other endocytic regulatory proteins to stimulate the formation of the clathrin coat [81]. In this model, the clathrin adaptor complex AP-2 plays a central role in CCP formation and function, being responsible for the assembly of clathrin triskelia at the plasma membrane and selection of cargo receptors that will be internalized by forming CCVs. AP-2 is a heterotetramer composed of two large subunits (*α*-adaptin and *β*2-adaptin), a medium subunit (*μ*2), and a small subunit (*σ*2). It has been known for some time that several proteins associated with clathrin-coated vesicles are substrates for protein kinases. These proteins include AP-2, dynamin, clathrin, synaptojanin 1, and amphiphysins. However, the identities of the kinases involved in this process remained largely unknown until recently. So far, only three of the four adaptor complex subunits of AP-2 have been shown to be phosphorylated. 

### 5.1. Serine/Threonine Phosphorylation of the *μ*2-Subunit of AP-2 by Cyclin G-Associated Protein Kinase (GAK)

It has been known for many years that *μ*2 is phosphorylated both *in vivo*, and *in vitro* but the functional relevance of these posttranslational modifications was unclear [82]. The identity of the kinase that phosphorylates *μ*2 remained obscure for a long time. Two candidate kinases have been described in the literature [83–88]. One of them, termed GAK (for cyclin G-associated protein kinase), was shown to phosphorylate *μ*2 recombinant proteins *in vitro* [86]. It was also demonstrated that GAK copurifies with CCVs, and that immunoprecipitated GAK can phosphorylate the endogenous *μ*2 subunit *in vitro* [85]. GAK is a complex protein; in addition to an N-terminal kinase domain, it contains a central tensin homology domain and a C-terminal J domain. This C-terminal domain shows similarity to auxilin, a neuronal protein that facilitates Hsc70 in the uncoating of CCVs. Hence, an alternative (and possibly more appropriate) name for GAK is auxilin 2 [87]. GAK was shown to bind directly to both clathrin and the appendage domain of *α*-adaptin [86]. GAK/auxilin 2 is localized mostly to the *trans*-Golgi network (in HeLa cells at least). However, its overexpression completely prevents transferrin uptake [86, 87, 89] implying a role for GAK/auxilin 2 in endocytosis. Given the multifunctional nature of GAK/auxilin 2, it is presently difficult to conclude whether the effect of GAK/auxilin 2 overexpression on endocytosis is due to its role in clathrin uncoating or *μ*2 phosphorylation.

### 5.2. Serine/Threonine Phosphorylation of the *μ*2-Subunit of AP-2 by *α*-Adaptin-Associated Kinase-1 (AAK1)

It was shown, *in vitro*, that *μ*2 phosphorylation is required for internalization of Transferrin receptor [90]. However, with the identification of Thr-156 as a residue essential for clathrin-coated pit function* in vivo* [83, 84], it appears clearly that phosphorylation play an important role in endocytosis. Several studies indicate that the phosphorylation of Thr-156 on *μ*2-subunit induced by *α*-adaptin-associated kinase-1 (AAK1), could regulate its binding to endocytic cargo [83, 84, 91]. AAK1 was identified using a phage display library screening strategy [84]. Just like GAK/auxilin 2, AAK1 is enriched in purified CCV preparations and phosphorylates the *μ*2 subunit of AP-2 *in vitro *[84]. Although some observations have demonstrated that the *μ*2 phosphorylation is not obligatory for receptor uptake, it is critical for maximizing internalization efficiency [90, 92]. Additionally, serine/threonine phosphorylation of AP-2 in the hinge region, between the C-terminal ear and the N-terminal domains of the beta-subunit, has been shown to affect AP-2's ability to interact with clathrin and to associate with membranes [74]. Thus, phosphorylation of AP-2 complexes regulates their recruitment to the plasma membrane [93], their interaction with cargo molecules containing tyrosine-sorting signals [83], and their assembly with clathrin [74, 94].

### 5.3. Phosphorylation of *α*- and *β*2-Subunits of AP-2 by CK2

It was found that Casein kinase 2 (CK2) phosphorylates the *α*− and *β*2-subunits of the AP-2 adaptor complex *in vitro* [85]. It is possible that CK2 plays the same role *in vivo*. *In vitro* phosphorylation of AP-2 by CK2 prevents AP-2 from binding to clathrin cages, thus mimicking the effect observed *in vivo* [74].

### 5.4. Tyrosine Phosphorylation of *β*2-Subunit of AP-2 by Src

The list of GPCRs-activated signaling pathways in which *β*-arrestins have been implicated includes not only components of the clathrin-endocytic machinery, but also signaling molecules such as the non-receptor tyrosine kinase Src family [95–101]. Src family kinases exhibit a highly conserved structural organization that includes a myristoylated N-terminal domain to facilitate its attachment at the plasma membrane, followed by the SH3 domain recognizing a proline-rich region, the SH2 domain recognizing tyrosine phosphorylated residues, the tyrosine kinase domain (SH1) and a C-terminal tail [102]. Thus, targeting of Src family kinase is accomplished through protein-protein interactions involving the phosphotyrosine-binding Src homology domain 2 (SH2) and proline-rich domain-binding SH3 domains common to all members of the family. Today, c-Src is described as an important regulator of GPCRs endocytosis based on MEF cells deficient of Src-family tyrosine kinases (SYF cells), where the internalization of *β*2AR was abolished [103]. Upon stimulation of the *β*2AR, c-Src rapidly associates with the receptor in a *β*-arrestin-dependent manner [62]. c-Src mutants devoid of catalytic activity that can interact with *β*-arrestin (SH1-KD) function as dominant-negative inhibitors of clathrin-mediated internalization of receptors [100, 104]. Also, several new substrates for the c-Src phosphorylation of proteins involved in the endocytic machinery have been identified (see Table 1).

It has been suggested that c-Src recruitment to the endocytic complexes may play a role in the activation of the endocytic machinery [78, 100, 101]. This has been confirmed by Fessart et al. that the recruitment of Src to the *β*-arrestin/AP-2 complex is necessary for the agonist-dependent phosphorylation of the *β*2-subunit on Tyr-737, and regulates the disassembly of the endocytic complex and AT1R internalization [105]. This mechanism has been demonstrated for other GPCRs like the *β*2-adrenergic, vasopressin V2, bradykinin type 2, platelet-activating factor, and endothelin A receptors but also for other type of receptors such as epidermal growth factor receptor [106].

## 6. Regulation of GPCRs Internalization by Dynamin Phosphorylation

During receptor internalization, it was initially thought that *β*-arrestin participated in the *de novo* formation of CCPs [107, 108]. However, recent evidence shows that *β*-arrestin targets receptors to existing pits at the cell surface and engage them in the internalization process [109, 110]. Although this issue remains controversial, the general model for receptor internalization from the plasma membrane is that the formed pits progressively invaginate and are finally released into the cytosol as free CCV, a step which requires the activity of dynamin. Dynamin is a GTPase involved in the pinching off of clathrin-coated vesicles from the plasma membrane [6].

### 6.1. Dynamin Phosphorylation by c-Src

Dynamin is regulated by tyrosine phosphorylation during *β*2-AR internalization. The proposed model is that *β*-arrestin will act as an adaptor/scaffold to bind c-Src, allowing the phosphorylation of dynamin [100]. Ahn and collaborators identified two tyrosines (Y231F/Y597F) in dynamin that, when mutated, cannot be tyrosine phosphorylated by c-Src, and thus inhibit the agonist-induced internalization of the *β*2-AR. These data were also confirmed for the M1 muscarinic receptor [111], suggesting that tyrosine phosphorylation of dynamin plays an important role in endocytosis. Recently, Src kinase activity has been shown to regulate the endocytosis of the transferrin by phosphorylating two important components of the endocytic machinery, namely, the large GTPase dynamin 2 (Dyn2) and its associated actin-binding protein, cortactin (Cort) [112]. Src phosphorylation of dynamin seems also to act to increase both dynamin self-assembly and GTPase activity [113]. 

### 6.2. Dynamin Phosphorylation by Protein Kinase C (PKC)

Dynamin I is also phosphorylated by protein kinase C *in vitro* and probably by PKC in intact nerve terminals [114–117]. Dynamin I is then dephosphorylated by the Ca^2+^-dependent phosphatase calcineurin *in vitro* and in nerve terminals [118–121]. After dephosphorylation, dynamin I is fully rephosphorylated within 2 min [114, 122]. Rephosphorylation can be inhibited with a range of low-specificity PKC antagonists, suggesting that PKC is the dynamin I kinase in nerve terminals [114]. This PKC-mediated phosphorylation of dynamin has been proposed as a mechanism to remove dynamin I from the plasma membrane to a cytosolic compartment, where it is appropriately localized to participate in the next cycle of endocytosis [123].

### 6.3. Dynamin Phosphorylation by Cyclin-Dependent Kinase 5 (Cdk5)

Although several kinases for dynamin have been reported based on *in vitro *studies, Cdk5 has recently been reported to phosphorylate dynamin both *in vivo *and *in vitro*, and the phosphorylation sites have been determined. Cdk5 is one of the proline-directed kinases and phosphorylates serine/threonine motifs [124]. Cdk5 phosphorylates Thr-780 in dynamin 1 [125]. The phosphorylation of dynamin reduces its ability to interact with the amphiphysin 1.

## 7. Regulation of GPCRs Internalization by Synaptojanin 1 Phosphorylation

Synaptojanin 1 is a presynaptic inositol 5-phophatase enriched on endocytic intermediates [126]. As mentioned previously, synaptojanin is also found to be phosphorylated *in vivo *[127], but the functional role of this phosphorylation event in endocytosis remains to be determined.

## 8. Regulation of GPCRs Internalization by Amphiphysin Phosphorylation

Amphiphysin dimer binds both dynamin and Synaptojanin 1 [128–130]. Disruption of these interaction blocks clathrin-mediated endocytosis at the step of invaginated coated pits [131]. The amphiphysin also binds to clathrin and to the *α*-subunit of AP-2 [131, 132]. The phosphorylation of amphiphysin affects its binding to AP-2. Thus, this complex formation is inhibited by phosphorylation [127]. At least 3 protein kinases have been shown to phosphorylate amphiphysin I: Cdk5, MAPK/ERK and CK2. 

### 8.1. Amphiphysin Phosphorylation by Cyclin-Dependent Kinase 5 (Cdk5)

Cdk5 has recently been reported also to phosphorylate amphiphysin 1 *in vivo *and *in vitro*, and the phosphorylation sites have been determined. Cdk5 phosphorylates Ser- residues 261, 272, 276, and 285 and Thr-310 in amphiphysin 1 [125]. The phosphorylation at these sites is important for regulating the interactions of amphiphysin I with its endocytic partners, clathrin, AP-2, endophilin and lipid membrane. Cdk5 has multiple functions in neurons, implicated in the regulation of a range of cellular processes from adhesion and motility to synaptic plasticity and drug addiction [124, 133]. Cdk5 is abundant in presynaptic terminals in mature neurons [134]. The simultaneous phosphorylation of both amphiphysin I and dynamin I inhibits synaptic vesicle endocytosis through inhibition of the association of these proteins with their partner proteins such as *β*-adaptin [125]. Therefore, it is possible that the phosphorylation directly regulates the intramolecular interaction in amphiphysin, which in turn regulates the interaction with dynamin.

### 8.2. Amphiphysin Phosphorylation by Mitogen-Activated Protein Kinase/Extra-Cellular-Signal-Regulated Kinase (MAPK/ERK)

As a second kinase, mitogen-activated protein kinase/extra-cellular-signal-regulated kinase has been shown to phosphorylate Amphiphysin I at two sites, also within the proline/arginine rich domain (PRD) but distinct from those for cdk5. MAPK/ERK phosphorylates amphiphysin I at Ser-285 and Ser-293. This phosphorylation negatively regulates the amphiphysin binding to AP-2 [135]. 

### 8.3. Amphiphysin Phosphorylation by CK2

Phosphorylation of amphiphysin-1 can regulate its interaction with the heavy chain of clathrin. This interaction is mediated by two clathrin binding motifs of amphiphysin, the clathrin box and the W box. Two threonine residues, conserved in amphiphysin-1 and -2, are among several vertebrate species that can be phosphorylated by CK2. The phosphorylation of Thr-350 and Thr-387 in amphiphysin by CK2 regulates the interaction of amphiphysin and clathrin [136].

## 9. Different Roles of Proteins Phosphorylation

Coated vesicle formation is a constitutive process that involves continuous cycling of the coat proteins from the cytosol onto the membrane. Reversible phosphorylation is, therefore, a plausible mechanism for how this might be regulated. Phosphorylation events can be classified into two functionally opposite classes: (i) those that are inhibitory for CCV formation and (ii) those that facilitate the assembly of CCVs.

Few examples of phosphorylation mechanisms occurring during GPCRs internalization were previously reported (see Table 1). Indeed, dynamin was shown to be tyrosine phosphorylated and to regulate *β*2-AR internalization [100]. Interestingly, Fessart et al. found that activation of AT1R promotes the tyrosine phosphorylation of the *β*2-subunit of AP-2, to regulate its interaction with *β*-arrestin [101, 105]. Probably another best characterized example is phosphorylation in the hinge region of the *α*- and *β*2-subunits of the AP-2 complex. Phosphorylated AP-2 has a reduced ability to interact with clathrin and requires dephosphorylation for efficient interaction [74, 77]. Phosphorylation also appears to be important in modulating the function of several other proteins implicated in endocytosis; these include dynamin 1, clathrin, amphiphysins 1 and 2, AP-180, synaptojanin, epsin and Eps15. These proteins are found to be phosphorylated in resting nerve terminals and dephosphorylated when stimulation invokes a burst of CCV formation [123]. It was shown that phosphorylation of dynamin 1 and synaptojanin 1 inhibits their binding to amphiphysin, while phosphorylated amphiphysin has an impaired affinity for AP-2 and clathrin [127]. For example, *in vitro* dephosphorylation of rat brain extracts apparently promotes the assembly of dynamin I, synaptojanin, amphiphysin, clathrin, and AP-2 into complexes [127]. 

The other angle of the story that would be particularly interesting is the *in vivo* roles of dissociation. In others words, how and why these endocytic complexes dissociate? Close examination of AP-1 [137] and *β*-arrestin [65] shows that an effective way of regulating compartmental specificity could be dephosphorylation or phosphorylation by phosphatases and kinases restricted to defined membrane regions. These focal posttranslational modifications could modulate the avidity between network members, promoting assembly (and disassembly) of sorting lattices only at the appropriate location and time. For example, it has been reported that Ark1p (standing for actin regulating kinase 1)/Prk1p (standing for p53 regulating kinase 1)-mediated phosphorylation of a *S. cerevisiae* endocytic, eps15-like accessory factor governs the post budding inactivation of Arp2/3-dependent actin polymerization, preparing the transport vesicle for subsequent fusion [138]. The role of protein phosphorylation in controlling clathrin-coated vesicle formation at the cell surface remains relatively unexplored. 

Another explanation is that phosphorylation could also promote the recruitment of new interacting proteins with a SH2 domain. Nevertheless, proof of this model will require crystallization of the phosphorylated endocytic complexes. For example, since the crystal structure was obtained for *β*2-adaptin [139], it will be important in future studies to examine the crystal structure of wild-type *β*2-adaptin as compared to phosphorylated *β*2-adaptin. A structural approach will be useful in demonstrating the ability of phosphorylated *β*2-adaptin to bind, or not, endocytic proteins. It is not unlikely that a protein with an SH2 domain would make an excellent binding partner for c-Src phosphorylated tyrosine residue within *β*2-adaptin. Other AP-2 subunits when phosphorylated increase their affinity for different proteins. The affinity of phosphorylated *μ*2 for peptides containing the tyrosine-containing internalization motif in the cytoplasmic tails of receptors has been shown to be much greater that of unphosphorylate *μ*2. Recent structural data on AP-2 suggest a possible molecular explanation for these observations. Apparently, the binding site in *μ*2 for the tyrosine-containing motif is buried in the AP-2 complex, thus a conformational change is required for its exposure. This could be triggered by phosphorylation of *μ*2 [140]. In parallel, to verify and identify new proteins interacting with phosphorylated endocytic proteins, a proteomic approach would inform us on the partner(s) recruited during GPCRs endocytosis. 

How endocytic phosphorylation cycles are regulated is an important question for the future. Further studies on regulatory inputs and control mechanisms will provide important insight into the events controlling receptor endocytosis.

## 10. Futures Prospects in Phosphorylation and Internalization

While the mechanisms involved in regulating the clathrin-mediated endocytic pathway are not fully understood, accumulating evidence suggests that phosphorylation cycles may be a key step [127, 141]. Indeed, phosphorylation of the clathrin heavy chain [78] and the large and medium subunits of AP-2 [74, 142] have been demonstrated to be important to the clathrin-mediated internalization of growth factors and nutrients, respectively. However, the phosphorylation of endocytic factors is not limited to the major coat components. Many others constituents of the endocytic machinery including dynamin1, amphiphysin 1 and 2, synaptojanin, AP180, epsin and eps15, and AP-2 are phosphorylated. Although it is clear that phosphorylation plays an important role in clathrin-mediated internalization, a more detailed understanding of the endocytic regulatory mechanisms will require the identification of the respective kinases that target endocytic components. Multiple kinases of clathrin-coated vesicles have been defined, for example AAK1 phosphorylates the *μ*2 subunit of AP-2 [84], Src kinase phosphorylates clathrin [78], and the *β*2-subunit of AP-2 [105]. Previous observations suggest that the poly-L-lysine dependent phosphorylation of AP-2 prevents its recruitment to clathrin cages [74]. Thus, it is reasonable to believe that phosphorylation may regulate the ability of AP-2 to interact with other endocytic proteins, such as *β*-arrestin. However, we can not exclude the fact that phosphorylation may also influence AP-2 localization. Previous studies have established that *β*2-adaptin is phosphorylated *in vivo* by a staurosporine-sensitive kinase whose function is balanced by the constitutive activity of protein phosphatase 2A (PP2A) [142]. They also observed that treatment of cells with agents that block PP2A function perturb AP-2 localization at the plasma membrane and disrupt transferrin internalization. Thus, as suggested by Slepnev et al. [127], it is possible that one potential mechanism to regulate the cycles of coat protein assembly and disassembly is reversible phosphorylation.

In conclusion, phosphorylation appears to be an important mechanism by which GPCRs regulate their own internalization. We currently have only a few pieces of the puzzle available. The identity of many kinases and phosphatases, their location, and the timing of their action remain to be determined. In the future, it will be interesting to investigate the molecular consequences of protein phosphorylation, how kinases are regulated, and where and how phosphorylation *versus* dephosphorylation occurs. These issues are crucial to our understanding for the regulation of receptor internalization through clathrin-coated vesicles.

## Figures and Tables

**Table 1:** Functional role of phosphorylation by Protein Kinase(s) *in vitro* and *in vivo* during clathrin-mediated internalization.

Phosphorylation substrates	Kinase	Possible roles	References
Dynamin [Tyr-231 Tyr-597]	Src		[100]
Dynamin	PKC	stimulates GTPase activity	[115]
Dynamin [Thr-780]	Cdk5	Inhibition of Amphiphysin binding	[125]
Angiotensin II type 1 receptor	Src		[143]
*β*2-AR, prostacyclin receptor	PKA	switch coupling from G_s_ to G_i_	[16, 17]
GPCRs [Ser/Thr] clusters	GRKs	Desensitization, recruitment of arrestins	[26]
AP-2 [*μ*(YxxΦ)]	AAK1	TfR endocytosis	[144]
AP-2 [*μ*(YxxΦ)]	AAK1	*α*1a AR endocytosis	[145]
AP-2	AAK1	binding to endocytic cargo	[83, 84]
AP-2 [*μ*]	GAK		[85, 86]
AP-2 [*α*]	CK2	Inhibition of clathrin binding	[74, 77]
AP-2 [*β*]	CK2	Inhibition of clathrin binding	[74, 77]
AP-2 [*β*] [Tyr-737]	c-Src	Inhibition of arrestin binding	[105]
GRK2	PKC	Increase GRK activity	[36, 38]
GRK5	PKC	Decrease GRK activity	[39]
GRK2	c-Src	targets GRK2 for degradation	[40, 41]
*β*-arrestin 1 [Ser-412]	ERK1,2	Reduce binding to Src and clathrin	[60, 61]
*β*-arrestin 1 [Ser-412]	GRK5	Block Src activation	[63]
*β*-arrestin 1 [Tyr-54]	Src	Decrease the interaction with the *μ*-adaptin	[72]
*β*-arrestin 2 [Thr-383, Ser-361]	casein kinase II	interaction with unidentified binding partners	[64][65]
Arr2	CaMKII	Unknown	[66]
Clathrin heavy chain	Src	Facilitates membrane binding	[78]
Clathrin Light chain	CK2	Unknown	[75]
Amphiphysin [Thr-350,-387]	CK2	CK2 inhibits interaction with AP-2 and clathrin	[127, 136, 146]
Amphiphysin [Ser-261, 272, 276, 285 and Thr-310]	Cdk5	Inhibits interaction with the beta-subunit of AP-2	[125]
Amphiphysin	MAPK/ERK	Inhibits interaction with AP-2	[135]
Synaptojanin	PKC	Inhibits interaction with binding partners	[123, 127]
Synaptotagmin	CaMKII	Probably inhibitory	[87]
Epsin	Unknown	Inhibits interaction with binding partners	[147]
Eps15	Unknown	Inhibits interaction with AP-2	[147]

## References

[1] DeWire SM, Ahn S, Lefkowitz RJ, Shenoy SK (2007). *β*-arrestins and cell signaling. *Annual Review of Physiology*.

[2] Shenoy SK, Lefkowitz RJ (2005). Seven-transmembrane receptor signaling through beta-arrestin. *Science*.

[3] Lefkowitz RJ, Rajagopal K, Whalen EJ (2006). New roles for beta-arrestins in cell signaling: not just for seven-transmembrane receptors. *Molecular Cell*.

[4] Ferguson SSG (2007). Phosphorylation-independent attenuation of GPCR signalling. *Trends in Pharmacological Sciences*.

[5] Samama P, Cotecchia S, Costa T, Lefkowitz RJ (1993). A mutation-induced activated state of the *β*-adrenergic receptor. Extending the ternary complex model. *Journal of Biological Chemistry*.

[6] Damke H, Baba T, Warnock DE, Schmid SL (1994). Induction of mutant dynamin specifically blocks endocytic coated vesicle formation. *Journal of Cell Biology*.

[7] Hausdorff WP, Caron MG, Lefkowitz RJ (1990). Turning off the signal: desensitization of *β*-adrenergic receptor function. *FASEB Journal*.

[8] Sibley DR, Strasser RH, Benovic JL (1986). Phosphorylation/dephosphorylation of the *β*-adrenergic receptor regulates its functional coupling to adenylate cyclase and subcellular distribution. *Proceedings of the National Academy of Sciences of the United States of America*.

[9] Ribas C, Penela P, Murga C (2007). The G protein-coupled receptor kinase (GRK) interactome: role of GRKs in GPCR regulation and signaling. *Biochimica et Biophysica Acta*.

[10] Mestek A, Hurley JH, Bye LS (1995). The human *μ* opioid receptor: modulation of functional desensitization by calcium/calmodulin-dependent protein kinase and protein kinase C. *Journal of Neuroscience*.

[11] Diviani D, Lattion AL, Cotecchia S (1997). Characterization of the phosphorylation sites involved in G protein-coupled receptor kinase- and protein kinase C-mediated desensitization of the *α*(1B)-adrenergic receptor. *Journal of Biological Chemistry*.

[12] Qiu Y, Law PY, Loh HH (2003). *μ*-opioid receptor desensitization: role of receptor phosphorylation, internalization, and resensitization. *Journal of Biological Chemistry*.

[13] Benovic JL, DeBlasi A, Stone WC, Caron MG, Lefkowitz RJ (1989). *β*-Adrenergic receptor kinase: primary structure delineates a multigene family. *Science*.

[14] Freedman NJ, Liggett SB, Drachman DE, Pei G, Caron MG, Lefkowitz RJ (1995). Phosphorylation and desensitization of the human *β*-adrenergic receptor. Involvement of G protein-coupled receptor kinases and cAMP-dependent protein kinase. *Journal of Biological Chemistry*.

[15] Pierce KL, Premont RT, Lefkowitz RJ (2002). Seven-transmembrane receptors. *Nature Reviews Molecular Cell Biology*.

[16] Daaka Y, Luttrell LM, Lefkowitz RJ (1997). Switching of the coupling of the *β*-adrenergic receptor to different g proteins by protein kinase A. *Nature*.

[17] Lawler OA, Miggin SM, Kinsella BT (2001). Protein kinase A-mediated phosphorylation of serine 357 of the mouse prostacyclin receptor regulates its coupling to G(s)-, to G(i)-, and to G(q)-coupled effector signaling. *Journal of Biological Chemistry*.

[18] Fairfax BP, Pitcher JA, Scott MGH (2004). Phosphorylation and chronic agonist treatment atypically modulate GABAB receptor cell surface stability. *Journal of Biological Chemistry*.

[19] Pontier SM, Lahaie N, Ginham R (2006). Coordinated action of NSF and PKC regulates GABA receptor signaling efficacy. *EMBO Journal*.

[20] Kuramoto N, Wilkins ME, Fairfax BP (2007). Phospho-dependent functional modulation of GABA(B) receptors by the metabolic sensor AMP-dependent protein kinase. *Neuron*.

[21] Wilden U, Kühn H (1982). Light-dependent phosphorylation of rhodopsin: number of phosphorylation sites. *Biochemistry*.

[22] Stadel JM, Nambi P, Shorr RG, Sawyer DF, Caron MG, Lefkowitz RJ (1983). Catecholamine-induced desensitization of turkey erythrocyte adenylate cyclase is associated with phosphorylation of the beta-adrenergic receptor. *Proceedings of the National Academy of Sciences of the United States of America*.

[23] Shichi H, Somers RL (1978). Light-dependent phosphorylation of rhodopsin. Purification and properties of rhodopsin kinase. *Journal of Biological Chemistry*.

[24] Benovic JL, Strasser RH, Caron MG, Lefkowitz RJ (1986). *β*-adrenergic receptor kinase: identification of a novel protein kinase that phosphorylates the agonist-occupied form of the receptor. *Proceedings of the National Academy of Sciences of the United States of America*.

[25] Hausdorff WP, Lohse MJ, Bouvier M, Liggett SB, Caron MG, Lefkowitz RJ (1990). Two kinases mediate agonist-dependent phosphorylation and desensitization of the beta 2-adrenergic receptor. *Symposia of the Society for Experimental Biology*.

[26] Premont RT, Inglese J, Lefkowitz RJ (1995). Protein kinases that phosphorylate activated G protein-coupled receptors. *FASEB Journal*.

[27] Chen CY, Dion SB, Kim CM, Benovic JL (1993). *β*-adrenergic receptor kinase. Agonist-dependent receptor binding promotes kinase activation. *Journal of Biological Chemistry*.

[28] Sterne-Marr R, Benovic JL (1995). Regulation of G protein-coupled receptors by receptor kinases and arrestins. *Vitamins and Hormones*.

[29] Lodowski DT, Pitcher JA, Capel WD, Lefkowitz RJ, Tesmer JJG (2003). Keeping G proteins at bay: a complex between G protein-coupled receptor kinase 2 and G*βγ*. *Science*.

[30] Premont RT, Macrae AD, Stoffel RH (1996). Characterization of the G protein-coupled receptor kinase GRK4: identification of four splice vaeiants. *Journal of Biological Chemistry*.

[31] Pitcher JA, Freedman NJ, Lefkowitz RJ (1998). G protein-coupled receptor kinases. *Annual Review of Biochemistry*.

[32] Willets JM, Challiss RAJ, Nahorski SR (2003). Non-visual GRKs: are we seeing the whole picture?. *Trends in Pharmacological Sciences*.

[33] Kim J, Ahn S, Ren XR (2005). Functional antagonism of different G protein-coupled receptor kinases for *β*-arrestin-mediated angiotensin II receptor signaling. *Proceedings of the National Academy of Sciences of the United States of America*.

[34] Ren XR, Reiter E, Ahn S, Kim J, Chen W, Lefkowitz RJ (2005). Different G protein-coupled receptor kinases govern G protein and *β*-arrestin-mediated signaling of V2 vasopressin receptor. *Proceedings of the National Academy of Sciences of the United States of America*.

[35] Pitcher JA, Tesmer JJG, Freeman JLR, Capel WD, Stone WC, Lefkowitz RJ (1999). Feedback inhibition of G protein-coupled receptor kinase 2 (GRK2) activity by extracellular signal-regulated kinases. *Journal of Biological Chemistry*.

[36] Winstel R, Freund S, Krasel C, Hoppe E, Lohse MJ (1996). Protein kinase cross-talk: membrane targeting of the *β*-adrenergic receptor kinase by protein kinase C. *Proceedings of the National Academy of Sciences of the United States of America*.

[37] Cong M, Perry SJ, Lin FT (2001). Regulation of membrane targeting of the G protein-coupled receptor kinase 2 by protein kinase A and its anchoring protein AKAP79. *Journal of Biological Chemistry*.

[38] Chuang TT, LeVine H, De Blasi A (1995). Phosphorylation and activation of *β*-adrenergic receptor kinase by protein kinase C. *Journal of Biological Chemistry*.

[39] Chuang TT, Paolucci L, De Blasi A (1996). Inhibition of G protein-coupled receptor kinase subtypes by Ca2+/calmodulin. *Journal of Biological Chemistry*.

[40] Penela P, Elorza A, Sarnago S, Mayor F (2001). *β*-arrestin- and c-Src-dependent degradation of G-protein-coupled receptor kinase 2. *EMBO Journal*.

[41] Elorza A, Penela P, Sarnago S, Mayor F (2003). MAPK-dependent degradation of G protein-coupled receptor kinase 2. *Journal of Biological Chemistry*.

[42] Wilden U, Hall SW, Kuhn H (1986). Phosphodiesterase activation by photoexcited rhodopsin is quenched when rhodopsin is phosphorylated and binds the intrinsic 48-kDa protein of rod outer segments. *Proceedings of the National Academy of Sciences of the United States of America*.

[43] Zuckerman R, Cheasty JE (1986). A 48 kDa protein arrests cGMP phosphodiesterase activation in retinal rod disk membranes. *FEBS Letters*.

[44] Oakleyt RH, Jewell CM, Yudt MR, Bofetiado DM, Cidlowski JA (1999). The dominant negative activity of the human glucocorticoid receptor *β* isoform. Specificity and mechanisms of action. *Journal of Biological Chemistry*.

[45] Qian H, Pipolo L, Thomas WG (2001). Association of *β*-arrestin 1 with the type 1A angiotensin II receptor involves phosphorylation of the receptor carboxyl terminus and correlates with receptor internalization. *Molecular Endocrinology*.

[46] Pals-Rylaarsdam R, Gurevich VV, Lee KB, Ptasienski JA, Benovic JL, Hosey MM (1997). Internalization of the m2 muscarinic acetylcholine receptor. Arrestin-independent and -dependent pathways. *Journal of Biological Chemistry*.

[47] Vishnivetskiy SA, Paz CL, Schubert C, Hirsch JA, Sigler PB, Gurevich VV (1999). How does arrestin respond to the phosphorylated state of rhodopsin?. *Journal of Biological Chemistry*.

[48] Oakley RH, Laporte SA, Holt JA, Barak LS, Caron MG (1999). Association of *β*-arrestin with G protein-coupled receptors during clathrin-mediated endocytosis dictates the profile of receptor resensitization. *Journal of Biological Chemistry*.

[49] Lohse MJ, Benovic JL, Codina J, Cargon MG, Lefkowitz RJ (1990). *β*-arrestin: a protein that regulates *β*-adrenergic receptor function. *Science*.

[50] Gurevich VV, Dion SB, Onorato JJ (1995). Arrestin interactions with G protein-coupled receptors. Direct binding studies of wild type and mutant arrestins with rhodopsin, *β*-adrenergic, and m2 muscarinic cholinergic receptors. *Journal of Biological Chemistry*.

[51] Malecz N, Bambino T, Bencsik M, Nissenson RA (1998). Identification of phosphorylation sites in the G protein-coupled receptor for parathyroid hormone. Receptor phosphorylation is not required for agonist-induced internalization. *Molecular Endocrinology*.

[52] Richardson MD, Balius AM, Yamaguchi K, Freilich ER, Barak LS, Kwatra MM (2003). Human substance P receptor lacking the C-terminal domain remains competent to desensitize and internalize. *Journal of Neurochemistry*.

[53] Murray SR, Evans CJ, Von Zastrow M (1998). Phosphorylation is not required for dynamin-dependent endocytosis of a truncated mutant opioid receptor. *Journal of Biological Chemistry*.

[54] Jala VR, Shao WH, Haribabu B (2005). Phosphorylation-independent *β*-arrestin translocation and internalization of leukotriene B receptors. *Journal of Biological Chemistry*.

[55] Kim OJ, Gardner BR, Williams DB (2004). The role of phosphorylation in D dopamine receptor desensitization: evidence for a novel mechanism of arrestin association. *Journal of Biological Chemistry*.

[56] Torrecilla I, Spragg EJ, Poulin B (2007). Phosphorylation and regulation of a G protein-coupled receptor by protein kinase CK2. *Journal of Cell Biology*.

[57] Hanyaloglu AC, Vrecl M, Kroeger KM (2001). Casein kinase II sites in the intracellular C-terminal domain of the thyrotropin-releasing hormone receptor and chimeric gonadotropin-releasing hormone receptors contribute to beta-arrestin-dependent internalization. *Journal of Biological Chemistry*.

[58] Gaudreau R, Gouill CL, Venne MH, Stankova J, Rola-Pleszczynski M (2002). Threonine 308 within a putative casein kinase 2 site of the cytoplasmic tail of leukotriene B receptor (BLT1) is crucial for ligand-induced, G-protein-coupled receptor-specific kinase 6-mediated desensitization. *Journal of Biological Chemistry*.

[59] Rebholz H, Nishi A, Liebscher S, Nairn AC, Flajolet M, Greengard P (2009). CK2 negatively regulates G*α* signaling. *Proceedings of the National Academy of Sciences of the United States of America*.

[60] Lin FT, Krueger KM, Kendall HE (1997). Clathrin-mediated endocytosis of the *β*-adrenergic receptor is regulated by phosphorylation/dephosphorylation of *β*-arrestin1. *Journal of Biological Chemistry*.

[61] Lin FT, Miller WE, Luttrell LM, Lefkowitz RJ (1999). Feedback regulation of *β*-arrestin1 function by extracellular signal-regulated kinases. *Journal of Biological Chemistry*.

[62] Luttrell LM, Ferguson SSG, Daaka Y (1999). *β*-arrestin-dependent formation of *β*2 adrenergic receptor-src protein kinase complexes. *Science*.

[63] Barthet G, Carrat G, Cassier E (2009). Beta-arrestin1 phosphorylation by GRK5 regulates G protein-independent 5-HT 4 receptor signalling. *EMBO Journal*.

[64] Kim YM, Barak LS, Caron MG, Benovic JL (2002). Regulation of arrestin-3 phosphorylation by casein kinase II. *Journal of Biological Chemistry*.

[65] Lin FT, Chen W, Shenoy S, Cong M, Exum ST, Lefkowitz RJ (2002). Phosphorylation of *β*-arrestin2 regulates its function in internalization of *β*-adrenergic receptors. *Biochemistry*.

[66] Kahn ES, Matsumoto H (1997). Calcium/calmodulin-dependent kinase II phosphorylates Drosophila visual arrestin. *Journal of Neurochemistry*.

[67] Matsumoto H, Kurien BT, Takagi Y (1994). Phosrestin I undergoes the earliest light-induced phosphorylation by a calcium/calmodulin-dependent protein kinase in Drosophila photoreceptors. *Neuron*.

[68] Alloway PG, Dolph PJ (1999). A role for the light-dependent phosphorylation of visual arrestin. *Proceedings of the National Academy of Sciences of the United States of America*.

[69] Byk T, Bar-Yaacov M, Doza YN, Minke B, Selinger Z (1993). Regulatory arrestin cycle secures the fidelity and maintenance of the fly photoreceptor cell. *Proceedings of the National Academy of Sciences of the United States of America*.

[70] Richard EA, Lisman JE (1992). Rhodopsin inactivation is a modulated process in Limulus photoreceptors. *Nature*.

[71] Scott K, Zuker C (1997). Lights out: deactivation of the phototransduction cascade. *Trends in Biochemical Sciences*.

[72] Marion S, Fralish GB, Laporte S, Caron MG, Barak LS (2007). N-terminal tyrosine modulation of the endocytic adaptor function of the *β*-arrestins. *Journal of Biological Chemistry*.

[73] Xiao K, Sun J, Kim J (2010). Global phosphorylation analysis of *β*-arrestin-mediated signaling downstream of a seven transmembrane receptor (7TMR). *Proceedings of the National Academy of Sciences of the United States of America*.

[74] Wilde A, Brodsky FM (1996). In vivo phosphorylation of adaptors regulates their interaction with clathrin. *Journal of Cell Biology*.

[75] Bar-Zvi D, Branton D (1986). Clathrin-coated vesicles contain two protein kinase activities. Phosphorylation of clathrin *β*-light chain by casein kinase II. *Journal of Biological Chemistry*.

[76] Bar-Zvi D, Mosley ST, Branton D (1988). In vivo phosphorylation of clathrin-coated vesicle proteins from rat reticulocytes. *Journal of Biological Chemistry*.

[77] Georgieva-Hanson V, Schook WJ, Puszkin S (1988). Brain coated vesicle destabilization and phosphorylation of coat proteins. *Journal of Neurochemistry*.

[78] Wilde A, Beattie EC, Lem L (1999). EGF receptor signaling stimulates SRC kinase phosphorylation of clathrin, influencing clathrin redistribution and EGF uptake. *Cell*.

[79] Cotlin LF, Siddiqui MA, Simpson F, Collawn JF (1999). Casein kinase II activity is required for transferrin receptor endocytosis. *Journal of Biological Chemistry*.

[80] Shih W, Gallusser A, Kirchhausen T (1995). A clathrin-binding site in the hinge of the *β*2 chain of mammalian AP-2 complexes. *Journal of Biological Chemistry*.

[81] Traub LM (2003). Sorting it out: AP-2 and alternate clathrin adaptors in endocytic cargo selection. *Journal of Cell Biology*.

[82] Smythe E (2002). Regulating the clathrin-coated vesicle cycle by AP2 subunit phosphorylation. *Trends in Cell Biology*.

[83] Ricotta D, Conner SD, Schmid SL, Von Figura K, Höning S (2002). Phosphorylation of the AP2 *μ* subunit by AAK1 mediates high affinity binding to membrane protein sorting signals. *Journal of Cell Biology*.

[84] Conner SD, Schmid SL (2002). Identification of an adaptor-associated kinase, AAK1, as a regulator of clathrin-mediated endocytosis. *Journal of Cell Biology*.

[85] Korolchuk VI, Banting G (2002). CK2 and GAK/auxilin2 are major protein kinases in clathrin-coated vesicles. *Traffic*.

[86] Umeda A, Meyerholz A, Ungewickell E (2000). Identification of the universal cofactor (auxilin 2) in clathrin coat dissociation. *European Journal of Cell Biology*.

[87] Greener T, Zhao X, Nojima H, Eisenberg E, Greene LE (2000). Role of cyclin G-associated kinase in uncoating clathrin-coated vesicles from non-neuronal cells. *Journal of Biological Chemistry*.

[88] Flute SR, Porro EB, Slepnev VI, Ochoa GC, Tsai LH, De Camilli P (2001). Amphiphysin 1 binds the cyclin-dependent kinase (cdk) 5 regulatory subunit p35 and is phosphorylated by cdk5 and cdc2. *Journal of Biological Chemistry*.

[89] Lee DW, Zhao X, Yim YI, Eisenberg E, Greene LE (2008). Essential role of cyclin-G-associated kinase (auxilin-2) in developing and mature mice. *Molecular Biology of the Cell*.

[90] Olusanya O, Andrews PD, Swedlow JR, Smythe E (2001). Phosphorylation of threonine 156 of the *μ*2 subunit of the AP2 complex is essential for endocytosis in vitro and in vivo. *Current Biology*.

[91] Conner SD, Schmid SL (2003). Differential requirements for AP-2 in clathrin-mediated endocytosis. *Journal of Cell Biology*.

[92] Motley AM, Berg N, Taylor MJ (2006). Functional analysis of AP-2 *α* and *μ*2 subunits. *Molecular Biology of the Cell*.

[93] Fingerhut A, Von Figura K, Höning S (2001). Binding of AP2 to sorting signals is modulated by AP2 phosphorylation. *Journal of Biological Chemistry*.

[94] Semerdjieva S, Shortt B, Maxwell E (2008). Coordinated regulation of AP2 uncoating from clathrin-coated vesicles by rab5 and hRME-6. *Journal of Cell Biology*.

[95] Luttrell LM, Hawes BE, Van Biesen T, Luttrell DK, Lansing TJ, Lefkowitz RJ (1996). Role of c-Src tyrosine kinase in G protein-coupled receptor- and G*βγ* subunit-mediated activation of mitogen-activated protein kinases. *Journal of Biological Chemistry*.

[96] Chen YH, Pouyssegur J, Courtneidge SA, Van Obberghen-Schilling E (1994). Activation of Src family kinase activity by the G protein-coupled thrombin receptor in growth-responsive fibroblasts. *Journal of Biological Chemistry*.

[97] Simonson MS, Herman WH (1993). Protein kinase C and protein tyrosine kinase activity contribute to mitogenic signaling by endothelin-1. Cross-talk between G protein-coupled receptors and pp60(c-src). *Journal of Biological Chemistry*.

[98] Schieffer B, Paxton WG, Chai Q, Marrero MB, Bernstein KE (1996). Angiotensin II controls p21 activity via pp60. *Journal of Biological Chemistry*.

[99] Ptasznik A, Traynor-Kaplan A, Bokoch GM (1995). G protein-coupled chemoattractant receptors regulate Lyn tyrosine kinase*·*Shc adapter protein signaling complexes. *Journal of Biological Chemistry*.

[100] Seungkirl A, Maudsley S, Luttrell LM, Lefkowitz RJ, Daaka Y (1999). Src-mediated tyrosine phosphorylation of dynamin is required for *β*-adrenergic receptor internalization and mitogen-activated protein kinase signaling. *Journal of Biological Chemistry*.

[101] Fessart D, Simaan M, Laporte SA (2005). c-Src regulates clathrin adapter protein 2 interaction with *β*-arrestin and the angiotensin II type 1 receptor during clathrin-mediated internalization. *Molecular Endocrinology*.

[102] Brown MT, Cooper JA (1996). Regulation, substrates and functions of src. *Biochimica et Biophysica Acta*.

[103] Huang J, Sun Y, Huang XY (2004). Distinct roles for Src tyrosine kinase in *β*-adrenergic receptor signaling to MAPK and in receptor internalization. *Journal of Biological Chemistry*.

[104] Miller WE, Maudsley S, Ahn S, Khan KD, Luttrell LM, Lefkowitz RJ (2000). *β*-arrestin1 interacts with the catalytic domain of the tyrosine kinase c-SRC. Role of *β*-arrestin1-dependent targeting of c-SRC in receptor endocytosis. *Journal of Biological Chemistry*.

[105] Fessart D, Simaan M, Zimmerman B (2007). Src-dependent phosphorylation of beta2-adaptin dissociates the beta-arrestin-AP-2 complex. *Journal of Cell Science*.

[106] Zimmerman B, Simaan M, Lee MH, Luttrell LM, Laporte SA (2009). c-Src-mediated phosphorylation of AP-2 reveals a general mechanism for receptors internalizing through the clathrin pathway. *Cellular Signalling*.

[107] Goodman OB, Krupnick JG, Santini F (1996). *β*-arrestin acts as a clathrin adaptor in endocytosis of the *β*2-adrenergic receptor. *Nature*.

[108] Zhang J, Barak LS, Winkler KE, Caron MG, Ferguson SSG (1997). A central role for *β*-arrestins and clathrin-coated vesicle-mediated endocytosis in *β*2-adrenergic receptor resensitization. Differential regulation of receptor resensitization in two distinct cell types. *Journal of Biological Chemistry*.

[109] Scott MGH, Benmerah A, Muntaner O, Marullo S (2002). Recruitment of activated G protein-coupled receptors to pre-existing clathrin-coated pits in living cells. *Journal of Biological Chemistry*.

[110] Santini F, Gaidarov I, Keen JH (2002). G protein-coupled receptor/arrestin3 modulation of the endocytic machinery. *Journal of Cell Biology*.

[111] Werbonat Y, Kleutges N, Jakobs KH, Van Koppen CJ (2000). Essential role of dynamin in internalization of M muscarinic acetylcholine and angiotensin AT(1A) receptors. *Journal of Biological Chemistry*.

[112] Cao H, Chen J, Krueger EW, McNiven MA (2010). Src-mediated phosphorylation of dynamin and cortactin regulates the "constitutive" endocytosis of transferrin. *Molecular and Cellular Biology*.

[113] Ahn S, Kim J, Lucaveche CL (2002). Src-dependent tyrosine phosphorylation regulates dynamin self-assembly and ligand-induced endocytosis of the epidermal growth factor receptor. *Journal of Biological Chemistry*.

[114] Robinson PJ (1992). Differential stimulation of protein kinase C activity by phorbol ester or calcium/phosphatidylserine in vitro and in intact synaptosomes. *Journal of Biological Chemistry*.

[115] Robinson PJ, Sontag JM, Liu JP (1993). Dynamin GTPase regulated by protein kinase C phosphorylation in nerve terminals. *Nature*.

[116] Liu JP, Powell KA, Sudhof TC, Robinson PJ (1994). Dynamin I is a Ca-sensitive phospholipid-binding protein with very high affinity for protein kinase C. *Journal of Biological Chemistry*.

[117] Sontag JM, Fykse EM, Ushkaryov Y, Liu JP, Robinson PJ, Sudhof TC (1994). Differential expression and regulation of multiple dynamins. *Journal of Biological Chemistry*.

[118] Liu JP, Sim ATR, Robinson PJ (1994). Calcineurin inhibition of dynamin I GTPase activity coupled to nerve terminal depolarization. *Science*.

[119] Nichols RA, Suplick GR, Brown JM (1994). Calcineurin-mediated protein dephosphorylation in brain nerve terminals regulates the release of glutamate. *Journal of Biological Chemistry*.

[120] Bauerfeind R, Takei K, De Camilli P (1997). Amphiphysin I is associated with coated endocytic intermediates and undergoes stimulation-dependent dephosphorylation in nerve terminals. *Journal of Biological Chemistry*.

[121] Marks B, McMahon HT (1998). Calcium triggers calcineurin-dependent synaptic vesicle recycling in mammalian nerve terminals. *Current Biology*.

[122] Robinson PJ, Liu JP, Powell KA, Fykse EM, Sudhof TC (1994). Phosphorylation of dynamin I and synaptic-vesicle recycling. *Trends in Neurosciences*.

[123] Cousin MA, Tan TC, Robinson PJ (2001). Protein phosphorylation is required for endocytosis in nerve terminals: potential role for the dephosphins dynamin I and synaptojanin, but not AP180 or amphiphysin. *Journal of Neurochemistry*.

[124] Dhavan R, Tsai LH (2001). A decade of CDK5. *Nature Reviews Molecular Cell Biology*.

[125] Tomizawa K, Sunada S, Lu YF (2003). Cophosphorylation of amphiphysin I and dynamin I by Cdk5 regulates clathrin-mediated endocytosis of synaptic vesicles. *Journal of Cell Biology*.

[126] McPherson PS, Garcia EP, Slepnev VI (1996). A presynaptic inositol-5-phosphatase. *Nature*.

[127] Slepnev VI, Ochoa GC, Butler MH, Grabs D, De Camilli P (1998). Role of phosphorylation in regulation of the assembly of endocytic coat complexes. *Science*.

[128] Wigge P, Köhler K, Vallis Y (1997). Amphiphysin heterodimers: potential role in clathrin-mediated endocytosis. *Molecular Biology of the Cell*.

[129] David C, McPherson PS, Mundigl O, De Camilli P (1996). A role of amphiphysin in synaptic vesicle endocytosis suggested by its binding to dynamin in nerve terminals. *Proceedings of the National Academy of Sciences of the United States of America*.

[130] Grabs D, Slepnev VI, Songyang Z (1997). The SH3 domain of amphiphysin binds the proline-rich domain of dynamin at a single site that defines a new SH3 binding consensus sequence. *Journal of Biological Chemistry*.

[131] Shupliakov O, Löw P, Grabs D (1997). Synaptic vesicle endocytosis impaired by disruption of dynamin-SH3 domain interactions. *Science*.

[132] Wang LH, Sudhof TC, Anderson RGW (1995). The appendage domain of *α*-adaptin is a high affinity binding site for dynamin. *Journal of Biological Chemistry*.

[133] Bibb JA, Chen J, Taylor JR (2001). Effects of chronic exposure to cocaine are regulated by the neuronal protein Cdk5. *Nature*.

[134] Tomizawa K, Ohta J, Matsushita M (2002). Cdk5/p35 regulates neurotransmitter release through phosphorylation and downregulation of P/Q-type voltage-dependent calcium channel activity. *Journal of Neuroscience*.

[135] Shang WH, Adachi Y, Nakamura A, Copeland T, Kim SR, Kamata T (2004). Regulation of amphiphysin1 by mitogen-activated protein kinase: its significance in nerve growth factor receptor-mediated endocytosis. *Journal of Biological Chemistry*.

[136] Döring M, Loos A, Schrader N, Pfander B, Bauerfeind R (2006). Nerve growth factor-induced phosphorylation of amphiphysin-1 by casein kinase 2 regulates clathrin-amphiphysin interactions. *Journal of Neurochemistry*.

[137] Ghosh P, Kornfeld S (2003). AP-1 binding to sorting signals and release from clathrin-coated vesicles is regulated by phosphorylation. *Journal of Cell Biology*.

[138] Toshima J, Toshima JY, Martin AC, Drubin DG (2005). Phosphoregulation of Arp2/3-dependent actin assembly during receptor-mediated endocytosis. *Nature Cell Biology*.

[139] Owen DJ, Vallis Y, Pearse BMF, McMahon HT, Evans PR (2000). The structure and function of the *β*2-adaptin appendage domain. *EMBO Journal*.

[140] Collins BM, McCoy AJ, Kent HM, Evans PR, Owen DJ (2002). Molecular architecture and functional model of the endocytic AP2 complex. *Cell*.

[141] Turner KM, Burgoyne RD, Morgan A (1999). Protein phosphorylation and the regulation of synaptic membrane traffic. *Trends in Neurosciences*.

[142] Lauritsen JPH, Menné C, Kastrup J, Dietrich J, Ødum N, Geisler C (2000). *β*2-Adaptin is constitutively de-phosphorylated by serine/threonine protein phosphatase PP2A and phosphorylated by a staurosporine-sensitive kinase. *Biochimica et Biophysica Acta*.

[143] Paxton WG, Marrero MB, Klein JD, Delafontaine P, Berk BC, Bernstein KE (1994). The angiotensin II AT receptor is tyrosine and serine phosphorylated and can serve as a substrate for the SRC family of tyrosine kinases. *Biochemical and Biophysical Research Communications*.

[144] Ohno H, Stewart J, Fournier MC (1995). Interaction of tyrosine-based sorting signals with clathrin-associated proteins. *Science*.

[145] Diviani D, Lattion AL, Abuin L, Staub O, Cotecchia S (2003). The adaptor complex 2 directly interacts with the *α*-adrenergic receptor and plays a role in receptor endocytosis. *Journal of Biological Chemistry*.

[146] Hao W, Luo Z, Zheng L, Prasad K, Lafer EM (1999). AP180 and AP-2 interact directly in a complex that cooperatively assembles clathrin. *Journal of Biological Chemistry*.

[147] Cremona O, Di Paolo G, Wenk MR (1999). Essential role of phosphoinositide metabolism in synaptic vesicle recycling. *Cell*.

